# A live vaccine to *Staphylococcus aureus* infection

**DOI:** 10.1080/21505594.2018.1426965

**Published:** 2018-03-21

**Authors:** Dane Parker

**Affiliations:** Department of Pediatrics, Columbia University , New York, NY, USA

**Keywords:** d-alanined, sepsis, staphylococcus aureus, vaccine

*Staphylococcus aureus* is an important human pathogen responsible for significant morbidity and mortality worldwide. *S. aureus* is a significant cause of bloodstream, skin and soft tissue infections and pneumonia. *S. aureus* can account for one fifth of all bloodstream infections [2]. Colonization with *S. aureus* is relatively common with up to 30% of the population being persistent carriers and is associated with increased risk of infection [3,4]. Recent reports by both the World Health Organization and Centers for Disease Control have highlighted the problem facing us due to antimicrobial resistance [5,6], with methicillin resistant *S. aureus* (MRSA) representing a major problem. MRSA infections have increased levels of mortality, hospital stay, septic shock and subsequent infections. MRSA infections account for over 94,000 cases and 18,000 deaths annually in the United States and while estimates of its economic impact vary, it accounts for billions of dollars in expenditure in the United States as well as other countries [7–9]. Given its importance, the development of a vaccine and new antimicrobials to *S. aureus* is of high importance.

There is no current vaccine to *S. aureus* infection. Studies in the past have relied on single antigen preparations, with current efforts weighted towards multiple antigens [10,11]. Past attempts have investigated such candidates as iron surface determinant A and capsular polysaccharides. Both showed excellent promise in mouse models however, did not replicate this success in human trials [12–16]. These results have questioned the utility of murine models to predict the importance of specific virulence factors in pathogenesis and hence the accuracy in vaccine development given the presence of human specific virulence factors in *S. aureus* [17–21]. *S. aureus* also produces protein A. Protein A is an abundant surface protein that is able to interact with the Fc portion of immunoglobulin, suppressing the adaptive immune response by limiting B cell antibody production [22]. Deletion or mutation of the IgG binding region of protein A to negate this effect has also shown promise in vaccine studies in mice [23], thus evidence indicates protein A is a hindrance to antibody production and action against *S. aureus*. These are some of the reasons why a *S. aureus* vaccine has not made it to market. *S. aureus* also expresses a large array of virulence factors such that a vaccine against any one may not prove effective and thus current trials are focused on multiple antigen preparations. These formulations contain a mixture of *S. aureus* virulence factors such as clumping factor A (ClfA), manganese transport protein C (MntC), fibronectin binding protein B (FnbB) and capsular polysaccharides and are in clinical trials currently [247–26]. The vaccines developed to date have all utilize purified proteins of *S. aureus* virulence factors or surface proteins and not attenuated live strains.

In this issue Moscoso *et al* [1] provide data using a novel vaccine candidate that uses a live *S. aureus* strain that is a D-alanine auxotroph of *S. aureus*. Only trace amounts of D-alanine are found in vertebrates and it is a major component of peptidoglycan in the cell wall of *S. aureus* [27]. They utilized a triple mutant lacking D-amino acid transaminase and two alanine racemase genes, both of which are involved in the synthesis of D-alanine. While no growth or morphological defects were observed in the presence of D-alanine, in its absence the mutant was unable to grow, causing cells to be of abnormal size and showing morphological evidence of cell wall disruption. In a model of lethal infection, the auxotroph was profoundly less virulent and was rapidly cleared from the blood and organs regardless of the route of administration: intravenous, intraperitoneal or catheter infection models. The auxotrophic mutant was shown to be protective to subsequent infection of the parenteral strains with 100% protection compared to no protection in unvaccinated controls. Consistent with this protection, bacterial counts in the kidneys, spleen, liver and lung were all significantly reduced. The utilization of this live vaccine approach was also confirmed in an immunocompromised model of infection using leukopenic mice treated with cyclophosphamide. The immune response was shown to consist of significantly high levels of IgG, IgG3 and IgM, with IgG and IgM titers increasing after the administration of a second vaccine dose. Pooled immune sera was also protective when transferred to naïve mice. Cross-reactive antibody titers were evident with several strains of *S. aureus* and the vaccine strain conferred protection to both a bovine strain and the highly prevalent USA300 strain in addition to the parental strain. An interesting observation is that while the auxotroph was rapidly cleared from mice, it was still able to induce the production of protective antibodies. While the data generated thus far is positive it remains to be seen how the live vaccine strategy will fare in other animal model systems, given the previous disconnects between murine and human studies. As *S. aureus* is a human-adapted pathogen, studies utilizing humanized mice or rabbits, both of which are susceptible to several human-specific virulence factors, will potentially solidify this strategy and determine its worth in pursuing this live vaccination strategy clinically.

This study makes an interesting and important contribution to the future development of preventative therapies to *S. aureus* infection. Both previous and current major vaccine efforts and trials to *S. aureus* have been using protein and polysaccharide subunit vaccines. This study highlights the potential use of a live attenuated strain of *S. aureus* in the prevention of this important pathogen.
